# Higher Language Ability is Related to Angular Gyrus Activation Increase During Semantic Processing, Independent of Sentence Incongruency

**DOI:** 10.3389/fnhum.2016.00110

**Published:** 2016-03-11

**Authors:** Helene Van Ettinger-Veenstra, Anita McAllister, Peter Lundberg, Thomas Karlsson, Maria Engström

**Affiliations:** ^1^Center for Medical Image Science and Visualization (CMIV), Linköping UniversityLinköping, Sweden; ^2^Department of Medical and Health Sciences, Linköping UniversityLinköping, Sweden; ^3^Department of Clinical and Experimental Medicine, Linköping UniversityLinköping, Sweden; ^4^Division of Speech and Language Pathology, CLINTEC, Karolinska InstitutetStockholm, Sweden; ^5^Radiation Physics, Department of Medical and Health Sciences, Linköping UniversityLinköping, Sweden; ^6^Radiology, Department of Medical and Health Sciences, Linköping UniversityLinköping, Sweden; ^7^Department of Behavioral Science and Learning and Linnaeus Centre HEAD, Linköping UniversityLinköping, Sweden

**Keywords:** fMRI, semantic processing, congruency, sentence reading, language ability, inferior frontal gyrus, angular gyrus

## Abstract

This study investigates the relation between individual language ability and neural semantic processing abilities. Our aim was to explore whether high-level language ability would correlate to decreased activation in language-specific regions or rather increased activation in supporting language regions during processing of sentences. Moreover, we were interested if observed neural activation patterns are modulated by semantic incongruency similarly to previously observed changes upon syntactic congruency modulation. We investigated 27 healthy adults with a sentence reading task—which tapped language comprehension and inference, and modulated sentence congruency—employing functional magnetic resonance imaging (fMRI). We assessed the relation between neural activation, congruency modulation, and test performance on a high-level language ability assessment with multiple regression analysis. Our results showed increased activation in the left-hemispheric angular gyrus extending to the temporal lobe related to high language ability. This effect was independent of semantic congruency, and no significant relation between language ability and incongruency modulation was observed. Furthermore, there was a significant increase of activation in the inferior frontal gyrus (IFG) bilaterally when the sentences were incongruent, indicating that processing incongruent sentences was more demanding than processing congruent sentences and required increased activation in language regions. The correlation of high-level language ability with increased rather than decreased activation in the left angular gyrus, a region specific for language processing, is opposed to what the neural efficiency hypothesis would predict. We can conclude that no evidence is found for an interaction between semantic congruency related brain activation and high-level language performance, even though the semantic incongruent condition shows to be more demanding and evoking more neural activation.

## Introduction

Semantic processing is an integral and important function in humans, necessary for reading, communication, as well as problem solving. Language processing under extrinsic constraints such as high task demands can both increase and decrease neural activation patterns, depending on the kind of demand that is imposed. Furthermore, intrinsic constraints such as the cognitive abilities of the participant may result in altered neural patterns. However, the research on how neural activity patterns during reading comprehension may change due to intrinsic or extrinsic constraints is inconclusive.

The “simple view of reading” is a model that identifies reading comprehension as a product of decoding skills (reading of single words) and language comprehension (such as interpreting sentences) (Hoover and Gough, 1990). For the current study, we were interested in the language comprehension component of reading comprehension, and in specific the semantic processing that is needed to comprehend language. Processing of semantic information is characterized by activation bilaterally in the temporal lobes and the angular gyri (Humphries et al., 2006), and it has been demonstrated that activation in the left inferior frontal gyrus (IFG) is coupled to the integration of sentence information (Hagoort, 2005). If the meaning of a read sentence is anomalous, an increase of activation is observed in frontal regions bilaterally and in left temporal regions (Shankweiler et al., 2008). Other neuroimaging methods also find temporal lobe involvment in semantic processing, such as when measuring event-related brain potentials. Consistently, an N400 effect emerges mainly from the left temporal lobe when meaningful semantic information is processed. This effect was more notably when particular unexpected semantic information was processed (Kutas and Federmeier, 2011). Interestingly, retrieval of semantic ambiguous words also activates the left temporal lobe (Snijders et al., 2009). In addition, the right IFG has shown to exhibit activation specific for semantic processing in contrast to syntactic processing (Kang et al., 1999; Kuperberg et al., 2000), and during processing of ambiguous sentences (den Ouden et al., 2015). However, right-hemispheric activation during semantic tasks is not consistently reported, and may sometimes be related to other processes such as decision making (Gan et al., 2013).

According to the simple view of reading, poor reading comprehension may be attributed to poor performance on one or both components (decoding and language comprehension). Poor reading ability is associated with increased activation in the IFG bilaterally during a semantic decision task (Chee et al., 2001; Weber et al., 2006), while activation in the left temporoparietal lobe was decreased (Weber et al., 2006). If we look at the interaction between extrinsic constraint modulations, a study on young adults (aged 16–24), in which one exchanged word changed the pragmatic meaning of the sentence towards anomalous, showed an increase of activation in the left IFG for higher-skilled readers (Shankweiler et al., 2008). In our previous studies on reading in relation to language ability in adults, we found that activation in language-related areas in the right-hemispheric IFG and temporal lobe was related to better language performance when participants were engaged in sentence reading (Van Ettinger-Veenstra et al., 2010, 2012). In these previous studies, we measured language performance including reading ability with a Swedish test battery to assess high-level language and found that there was an overlap of regions correlating to high performance on this high-level language assessment and high performance on a reading ability test (Van Ettinger-Veenstra et al., 2010). Furthermore, lower performance on this high-level language assessment has shown previously to correlate with self-reported language problems and working memory decline (Laakso et al., 2000). Poor language comprehension can be attributed to decreased semantic and syntactic comprehension, even when phonological decoding is intact, according to the simple view of reading. Moreover, high-level language functions such as drawing inferences from a text are affected in poor language comprehenders, a possible explanation would be underlying working memory problems (Catts et al., 2006). Working memory capacity is also a direct predictor of reading comprehension development (Seigneuric and Ehrlich, 2005).

The interaction between task demand and individual performance on working memory and reading ability tasks on brain activation was explored during processing of sentences with different syntactic complexity (Prat et al., 2007; Prat and Just, 2011). The authors found that individuals with higher working memory capacity or better reading capability had differentiating neural patterns during low- and high-demand conditions. These neural patterns were identified as neural efficiency and neural adaptability. The neural efficiency hypothesis states that cognitive networks work in a more efficient manner in intelligent brains, and therefore less task-specific activation is observed (Haier et al., 1992). This is more pronounced during tasks of moderate demand as these task may be differentiating for high and low performing participants. High performing participants may find a moderate demand task not that cognitively demanding and have the possibility to process the information in a more efficient and less neural energy consuming way (Neubauer and Fink, 2009). Indeed, participants with a large vocabulary or high working memory capacity exhibited more neural efficiency in task-specific regions (Prat et al., 2007; Prat and Just, 2011). Neural adaptability, in contrast, is the recruitment of additional brain regions outside of task-specific regions to solve a task. This additional recruitment increases when task demands get high, and especially so for participants with high working memory capacity, however there was no correlation observed with high vocabulary (Prat and Just, 2011). The authors suggest that neural efficiency is reflecting individual differences related to language, while neural adaptability may reflect more general cognitive processing abilities.

The neural relation between the semantic rather than syntactic congruency of sentences and language performance has, to the best of our knowledge, thus far not been researched in an adult population. Moreover, there is a scarcity of research on language proficiency in the Swedish language. Our previous findings indicated that semantic processing in the form of sentence reading evoked neural adaptability in the form of increased right-hemispheric activation in IFG and temporal lobe related to high language performance on a reading ability test and more so on a high-level language ability test (Van Ettinger-Veenstra et al., 2010, 2012). We used the high-level language ability assessment as performance measure because of this correlation with neural adaptability activation, and because of the discussed correlate with working memory; the latter was repeatedly found to be involved in language comprehension (Seigneuric and Ehrlich, 2005; Catts et al., 2006; Prat and Just, 2011).

The aim of our study was to investigate whether processing of congruent or incongruent sentences would evoke similar right-hemispheric contributing activity in relation to high-level language ability as observed in our previous studies (Van Ettinger-Veenstra et al., 2010, 2012), or reduced activation in task-specific regions as observed in relation to syntactic congruency (Prat et al., 2007; Prat and Just, 2011). This was investigated by administering a sentence reading task in healthy adults during functional magnetic resonance imaging (fMRI).

During incongruency processing, we would expect an increase in activation in classic language areas in the left-hemispheric IFG and temporal lobe because of the semantically incongruent sentences being more complex and demanding than processing of semantic congruent sentences.

We expected that high performance on the high-level language ability assessment would be characterized by more efficient functioning of—and thus less activity in—classic language areas in the left-hemispheric IFG and temporal lobe. Moreover, high performance would be characterized by recruitment of supporting language regions such as right-hemispheric IFG and temporal lobe, which may reflect neural adaptability.

## Materials and Methods

### Participants

Twenty-seven healthy adults, 14 female and 13 male, participated in this study after recruitment by advertisement (mean age 25.5 years, range 18–35 years). All participants except one were right-handed. We ensured that the left-handed participant exhibited left-dominant brain activation during the language tasks by calculating the laterality index using the same procedure as described in our previous work (Van Ettinger-Veenstra et al., 2010). Handedness was measured with the Edinburgh handedness inventory. Inclusion criteria were Swedish as the first language and a minimum of 9 years of schooling (elementary school). Exclusion criteria were self-reported language dysfunction including dyslexia, concomitant medical, neurological or psychiatric illness, use of psychoactive drugs, or contraindication for MRI. Ethical approval for the study was obtained from the Regional Ethical Review Board in Linköping (2010/157-31 and M152-07 T22-09, granted to ME). The participants provided their verbal and written informed consent to participate in this study.

### Cognitive Language Tasks

We assessed language ability with a version of the BeSS tests (*Bedömning av Subtila Språksvårigheter—Assessment of subtle language disabilities*; Laakso et al., 2000; Berg et al., 2003). The BeSS test has been developed as an in-depth assessment of patients with minimal to mild language problems. The BeSS test version used in this study consists of seven sub-tasks investigating the following abilities: repetition of long sentences, recreating sentences (sentence construction), making inferences (text understanding), comprehension of complex embedded (logico-grammatical) sentences, comprehension of ambiguous (garden-path) sentences, comprehension of metaphors, and word definitions (vocabulary). Each subtest of 10 questions can result in a maximum of 30 points, with a total of 210 points for the entire test, indicating the highest performance. The responses are given orally. A more detailed description can be read in Berg et al. (2003).

### MRI-Data Acquisition

The fMRI-images were obtained with the use of a gradient-echo planar imaging sequence, which is sensitive to the blood oxygen level dependent (BOLD) response. Repetition time (TR) = 3 s, time to echo (TE) = 40 ms, flip angle (FA) = 90°, voxel size = 3.0 × 3.0 × 3.0 mm^3^, slice gap = 0.5 mm, 35 slices, field of view (FOV) = 228 × 204 × 122 mm^3^. The slices were aligned between the floor of the *sella turcica* and the posterior angle of the fourth ventricle. The structural MRI-images were obtained with a Philips Achieva 1.5 T scanner, and an anatomical 3D T1-weighted image of each participant’s whole brain was acquired for normalizing the functional images (TR = 25 ms, TE = 4.6 ms, FA = 30°, voxel size = 1 × 1 × 1 mm^3^, 175 slices, FOV = 240 × 240 × 175 mm^3^).

The fMRI paradigm was presented with the use of high-resolution video goggles (Resonance Technology Inc., CA, USA). Task responses (button presses) were recorded with the use of a response box (LUMItouch, Photon Control Inc., Burnaby, BC, Canada). Superlab Pro 4 (Cedrus Corp., San Pedro, CA, USA) software was used for task presentation.

### fMRI Paradigm

**Figure 1:**
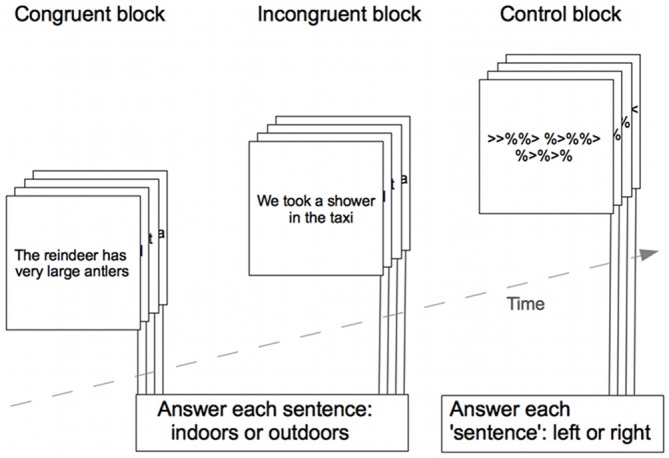
**Schematic outline of the paradigm used to assess semantic processing during fMRI.** The figure shows one example trial from each condition block, the actual sentences were in Swedish. Responses were given during each trial presentation.

Semantic processing was assessed by a visually presented sentence-reading paradigm, presented in a block design with three different blocks containing congruent sentences, incongruent sentences, or a baseline condition (see Figure 1). Congruent sentences represented actions or descriptions that were semantically meaningful, incongruent sentences represented actions or descriptions that were semantically non-plausible or nonsense. Sentences were taken from the Daneman and Carpenter listening span task (Daneman and Carpenter, 1980), and the sentences in the two different conditions were controlled for sentence length and familiarity of words based on occurrence on a Swedish word frequency list (Allén, 1973). The baseline condition had the visual appearance of a sentence, but consisted instead of an alternating number of “%” symbols and arrows, the arrows were all pointing either to the left or to the right.

Each block in the paradigm contained four trials and each trial lasted 3 s, then the next trial started immediately. Answers were to be given during the 3 s trial presentation time. There was a 1 s break between blocks. Presentation of the three different blocks (congruent, incongruent and baseline) was repeated 10 times, and block order was randomized within each repetition. The total paradigm length was approximately 6 min.

After each sentence, the participants were asked to respond using button presses on a response box to indicate whether the action or description took place indoors or outdoors. We did not process the indoor/outdoor responses any further, as the questions’ sole purpose was to ensure that the participants processed the sentences semantically during reading. In the baseline condition the participants were asked to indicate whether all arrows were pointing to the left or to the right. In this study, we used the participants’ responses as indicators of active participation in the task. Reaction times were collected during stimulus presentation time (3 s) and very fast responses (<400 ms) were discarded.

### Image Analysis

Preprocessing and analysis of the fMRI data were performed using SPM8^1^. During preprocessing, the fMRI images were realigned to the first image to correct for movement during scanning. Then, the images were co-registered to the participants structural MRI, normalized to a 2 × 2 × 2 mm^3^ Montreal Neurological Institute (MNI) template with the use of the parameters obtained from a segmentation of the structural MRI, and finally smoothed with an 8 mm full width at half maximum (FWHM) Gaussian kernel to reduce the effects of inter-participant differences in brain anatomy and to reduce noise levels. For the analysis of individual data, a general linear model was applied with a box-car modeling of the three condition blocks, and the effects of interest were convolved with the hemodynamic response function and corrected for movement by including the movement parameters as covariates to create individual statistical parametric maps for the three conditions.

### Statistical Tests

We investigated on a group level how neural activation upon semantic processing was related to congruent sentence reading, incongruent sentence reading, or to the difference between incongruent and congruent sentence reading processes. First, we explored neural activation distributed over the whole brain during semantic processing of congruent or incongruent sentence with one-sample *t*-tests, by applying the contrasts (congruent sentences) > (baseline) and (incongruent sentences) > (baseline). Results were regarded as significant corrected for multiple comparisons with a “Family Wise Error” rate (FWE) corrected voxel-wise *p*-value of *p* < 0.05.

**Figure 2:**
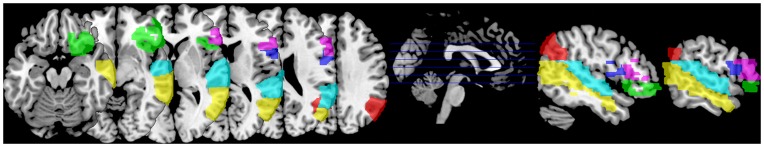
**Distribution of regions of interest (ROI; defined as Brodmann areas (BA), only right-hemispheric regions are shown) used in this study located in the inferior frontal gyrus (IFG); BA 44 pars opercularis (dark blue), BA 45 pars triangularis (purple), and BA 47 pars orbitalis (green), and in the temporoparietal region; BA 39 angular gyrus (red), BA 22 superior temporal gyrus (cyan) and BA 21 middle temporal gyrus (yellow)**.

For our subsequent group analyses, we were interested in neural activation within pre-defined regions of interests (ROIs) in language regions. We defined 12 ROIs based on the Brodmann atlas in the Wake Forrest University (WFU) Pickatlas (Maldjian et al., 2003) in the frontal, temporal, and parietal lobes bilaterally (see Figure 2). A dilation factor of three voxels was applied to the ROIs in order to account for the smoothing of the activation during preprocessing. Three ROIs were located in the IFG: Brodmann areas (BA) 44 or pars opercularis (cluster size left: 522 voxels, right: 452 voxels), BA 45 or pars triangularis (cluster size left: 896 voxels, right: 644 voxels), and BA 47 or pars orbitalis (cluster size left: 1743 voxels, right: 1147 voxels). Three ROIs were located in the temporoparietal lobe: BA 39 or angular gyrus (cluster size left: 1772 voxels, right: 1147 voxels), BA 22 or superior temporal gyrus (cluster size left: 2386 voxels, right: 1826 voxels), and BA 21 or middle temporal gyrus (cluster size left: 1719 voxels, right: 1538 voxels).

We then investigated brain activation that was modulated by semantic incongruency by comparing incongruent with congruent sentence reading with a one-sample *t*-test, using the (incongruent sentences) > (congruent sentences) contrast. We initially thresholded the contrast at *p* = 0.001, and applied small volume correction analyses, using the pre-defined ROIs. We report the results of these small volume correction analyses that were significant at *p* < 0.05, FWE corrected.

We applied a multiple regression analysis to investigate the relation of BeSS language ability performance scores and neural activation during semantic processing for the contrasts (congruent sentences) > (baseline) and (incongruent sentences) > (baseline). Since it is known that age may influence brain activation during language tasks, we controlled for age by including it as a covariate. As before, results exceeding a threshold of *p* = 0.001 were entered into a small volume correction analysis within the pre-defined ROIs, reported results were significant at *p* < 0.05, FWE corrected.

Lastly, we investigated differences between reaction times on the three semantic processing tasks with a repeated-measures analysis of variance (ANOVA). To investigate the relation between reaction times on the three fMRI semantic processing tasks, BeSS performance scores and age, we performed Pearson’s *r* correlations. This resulted in seven correlation analyses (three for reaction times correlated with BeSS performance score, three for reaction times and age, and one for BeSS and age). Therefore a Bonferroni-adjusted significance threshold of *p* < 0.007 was calculated to achieve a corrected significance of *p* < 0.05 for these correlations.

## Results

### Cortical Responses to Sentence Reading

**Figure 3:**
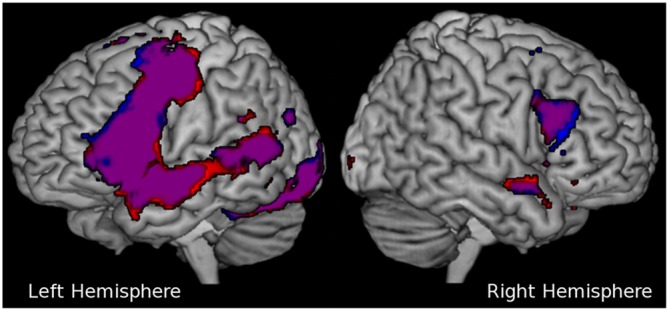
**Cortical activation in language areas during sentence reading.** The results of the group activation in both hemispheres during congruent sentence reading are shown in red, activation during incongruent sentence reading is shown in blue. The violet shaded areas show overlap in activation between the two conditions. For visualization purposes only, the activation in this figure was thresholded at *p* = 0.001, significant results are presented in Table 1.

Significant cortical activation during reading of congruent sentences was observed in the following regions (cluster peak values surviving a whole-brain analysis FWE-corrected and an extended voxel threshold of five are reported): left frontal lobe including the IFG right middle/IFG, left middle temporal gyrus, left fusiform gyrus, left lingual gyrus, and bilateral anterior cingulate cortex (predominantly in the left hemisphere) continuing to the supplementary motor area (SMA; see Table 1). These cortical areas are frequently attributed to the language network in the human brain (Bookheimer, 2002; Price, 2012). Figure 3 shows that cortical activation during incongruent sentence reading overlapped greatly with activation during congruent sentence reading.

**Table 1:** **Cortical activation during reading**.

Side	Activated region	Size	*Z*	*x*	*y*	*z*
Left	Frontal lobe (including inferior frontal gyrus)	1946	6.52	−44	−8	58
Right	Middle/inferior frontal gyrus	7	5.09	46	18	30
Right	Middle/inferior frontal gyrus	5	5.06	38	36	−14
Left	Middle temporal gyrus	567	6.30	−58	−36	2
Left	Fusiform gyrus	1223	7.00	−42	−48	−20
Left	Lingual gyrus	144	6.00	−12	−50	2
Bilateral	Anterior cingulate cortex/supplementary motor area	547	5.85	6	20	42

*The table shows the regions that were significantly activated during congruent sentence reading. Side, Activated brain hemisphere; BA, Brodmann Area; Size, Cluster size in voxels; Z, Z-value of the peak activation in the activated cluster. x, y and z are the peak coordinates in Montreal Neurological Institute (MNI) space. All activations were significant for a whole-brain analysis with a p < 0.05, Family Wise Error (FWE)-corrected*.

### Cortical Activation Correlated to Semantic Incongruency

**Figure 4:**
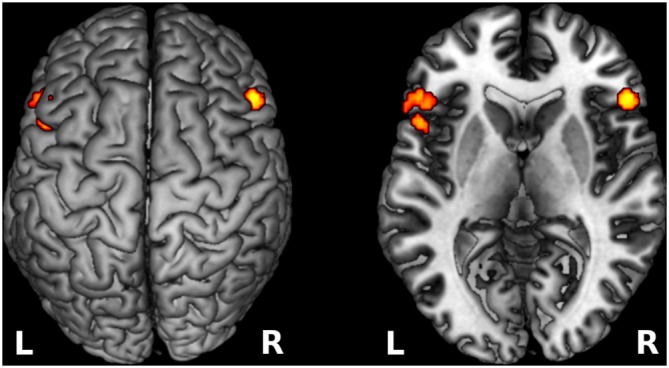
**Increased cortical activation for semantic incongruency contrasted with semantic congruency.** The figure shows activation in the IFG bilaterally (BA 45) as projected on the cortical surface (left), and the exact locus of activation visible on a transverse cutout slice (right). The visualized results refer to cortical activation that was more prominent during incongruent sentence reading compare to congruent sentence reading. L, left hemisphere; R, right hemisphere.

Data showed that the participants elicited increased activation in the IFG, specifically in BA 45 bilaterally during incongruent sentence reading compared to congruent sentence reading. Small volume correction analyses revealed significantly activated clusters with peak activation at MNI coordinates = (−50, 28, 6), *Z*-score = 4.1, cluster size = 896 voxels, and MNI coordinates = (52, 26, 24), *Z*-score = 4.75, cluster size = 644 voxels, respectively (Figure 4).

### Behavioral Results

The participants had a mean total score of 161.8 on the BeSS test (standard deviation (SD) = 21.4; range = 117–193), out of a maximum of 210.

**Figure 5:**
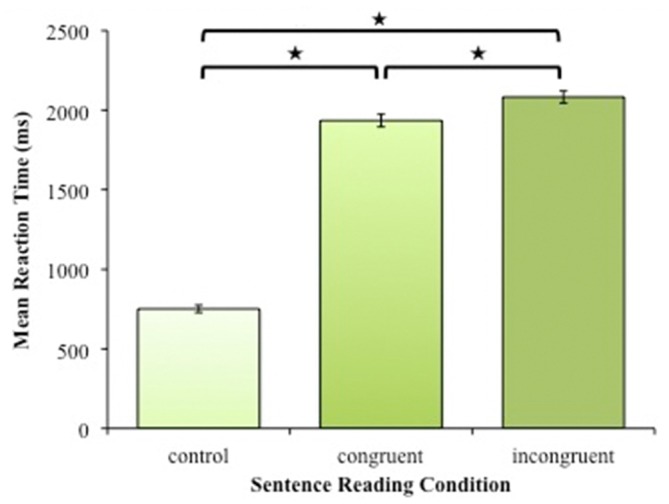
**Reaction times per condition during the sentence reading fMRI task.** The means are averaged across participants, the error bars represent the standard error of the mean. All means were significantly different from each other as indicated by an “*”.

We found that the reaction times during the three conditions differed significantly from each other at the level of *p* < 0.0001 (*F*_(2,26)_ = 666), *post hoc* Tukey’s range test showed significant differences between all pairs of group means at the level of *p* < 0.01. This indicates that the participants were slower in answering during the incongruent sentence condition (mean = 1947, SD = 216) than during the congruent condition (mean = 1811, SD = 242), and both sentence reading conditions were executed slower than the baseline condition (mean = 694 ms, SD = 81; Figure 5).

For our correlation analysis of reaction times, BeSS scores and age, we applied a Bonferroni-corrected threshold of *p* < 0.007. No significant correlations of reaction times with BeSS performance scores were observed. Neither did the reaction times, nor BeSS performance score correlate significantly with age, even though a trend was seen for the correlation between baseline reaction time and age (Pearson’s *r* = 0.48, *p* = 0.011 uncorrected).

### Cortical Activation and Language Ability

**Figure 6:**
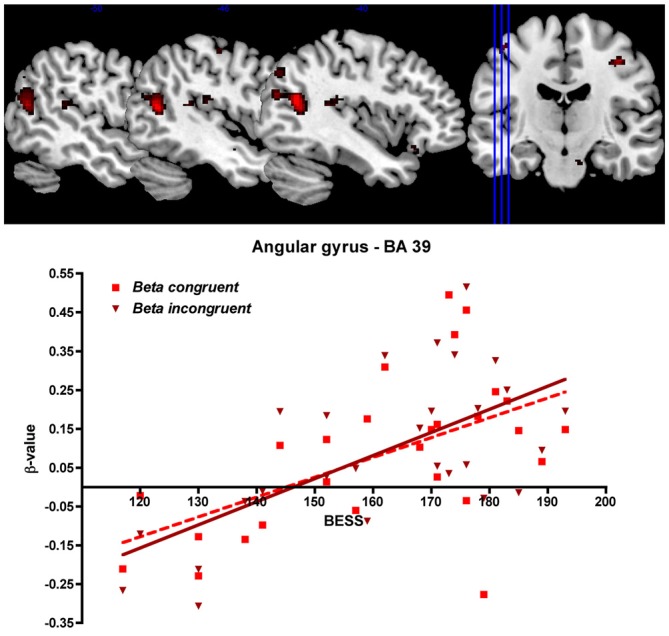
**Top**: Cortical activation related to language ability. The figure shows cortical activation in the left angular gyrus/temporal lobe during congruent sentence reading that was correlated to high-level language ability measured as high performance on the BeSS test. For visualization purposes, the threshold is set to *p* < 0.001 uncorrected in this image. Statistical data of the significant, corrected results are shown in Table 2. **Bottom**: Correlation between BeSS scores and the mean beta values (parametric estimates) from the region of interest encompassing left angular gyrus (for congruent respective incongruent sentence reading).

To investigate how language ability modulates cortical activation we entered the BeSS total scores as regressors in the analysis. In this way, we tested for the interaction between language ability scores and brain activation in our predefined ROIs. We found that cortical activation in the left angular gyrus (BA 39), continuing to the left superior temporal gyrus (BA 22) was increased during both congruent and the incongruent sentence reading when language ability was higher, measured by the BeSS test (see Table 2 and Figure 6).

**Table 2:** **Regression analysis between high-level language ability and cortical activation**.

Condition	Side	Activated region	BA	Size	*Z*	*x*	*y*	*z*
**Positive correlation between cortical activation and language ability**
Congruent	Left	Angular gyrus	39	51	4.57	−44	−62	18
					4.51	−40	−62	20
	Left	Superior temporal gyrus	22	31	4.37	−44	−60	14
Incongruent	Left	Angular gyrus	39	156	5.08	−38	−58	16
					4.83	−40	−62	20
	Left	Superior temporal gyrus	22	84	5.08	−38	−58	16
**Negative correlation between cortical activation and language ability**
n.s.

*The table shows the results from the correlation between BeSS test performance scores and cortical activation during congruent and incongruent sentence reading conditions, respectively. There were no significant negative correlations with brain activation and BeSS performance scores. Side, Activated brain hemisphere; BA, Brodmann Area; Size, Cluster size in voxels; Z, Z-value of the peak activation in the activated cluster. Coordinates x, y and z are in Montreal Neurological Institute (MNI) space. n.s., not significant. All activations were significant after small volume correction at p < 0.05, Family Wise Error (FWE)-corrected*.

## Discussion

In this study, we aimed to investigate whether increased performance on high-level language assessment is characterized by a corresponding neural activation increase or decrease in language-related regions during sentence reading. Furthermore, we investigated whether observed neural activation patterns are modulated by semantic incongruency.

### Cortical Activation and Semantic Incongruency

We expected that the difference between incongruent sentences vs. congruent sentences would evoke an increase of brain activation in classic cortical language areas. When comparing semantic incongruency with semantic congruency, we found increased activation in the IFG bilaterally for the whole group. This finding is in accordance with previous research on brain activation on syntactic complexity (Just et al., 1996; Prat et al., 2011) and imagery modulation (Cabeza and Nyberg, 2000; Yang et al., 2009), and it shows that the incongruent sentence condition was regarded as a more difficult task by the participants. This is supported by the difference in reaction times, showing that responses during the incongruent condition were on average slower than during the congruent condition. Interestingly, an fMRI study investigating the interpretation of incongruent sentences has also found activity in BA 45/47 upon contrasting incongruent to congruent sentences, however this was limited to the left hemisphere (Hagoort et al., 2004). The left IFG is important for unification of sentence information, both syntactical and—as above study shows—semantic. Importantly, the inferior frontal bilateral activation increase observed in the current study was restricted to BA 45 and did not extend into BA 47. Although our measures of language *ability* using the BeSS test are equivalent in both our current and previous studies, the *semantic* fMRI paradigms we used were different in terms of type of semantic processing (sentence completion in our previous study and sentence reading and drawing inferences in the current).

### Cortical Activation and Language Ability

Our main interest in this study was to investigate how performance on a high-level language assessment (BeSS) administrated prior to fMRI correlates to brain activation during semantic processing. A high score on the language assessment showed to correlate to increased activation in the left angular gyrus (BA 39) extending to the superior temporal gyrus (BA 22). This correlation was independent of semantic congruency, as it emerged for both the congruent and the incongruent condition, unlike findings in previous studies. Without correlation to language ability, semantic incongruency and ambiguous sentences were previously found to evoke more activation in the angular gyrus compared to congruent or unambiguous sentences (Newman et al., 2001; Tyler et al., 2011). The angular gyrus has different functionalities, related to anatomical subdivisions (Seghier, 2013), of which some seem to be specific for semantic processing such as comprehensive reading of sentences (Xu et al., 2005) and the angular gyrus is thought to be essential for comprehension of speech (Hartwigsen et al., 2015). Meyler et al. (2007, 2008) previously showed performance-dependent modulation of activation in the left angular gyrus in children. The authors found that performance modulation was dependent not only on reading capability, but also on age. They also reported that incapable readers under-activated the left angular gyrus, indicating that poor readers have less neural resources for comprehension of written material. With regard to the extension of the activation from the angular gyrus towards the temporal lobe in our study, Pallier et al. (2011) found that the angular gyrus was only activated in a word/pseudoword test when semantic information was present, however the posterior superior temporal sulcus was not activated. Interestingly, the origin of N400 effects are consistently found in the left temporal lobe as well as the angular gyrus upon processing meaningful information (Kutas and Federmeier, 2011). The N400 is not a static event but rather represents a wave of activity. However, because of the temporal limits of fMRI, this activity wave cannot be represented in the BOLD signal that we measure. In the light of these studies, we argue that the performance-dependent increase of activation in the angular gyrus may reflect the depth of semantic information processing. The participants with higher language ability may have a better or deeper processing of the meaning of each sentence. The nature of this increased processing of information could also be related to figurative language, as the most ventral part of the angular gyrus, corresponding to the activation we have found, has shown to be sensitive for visual inputs and theory of mind judgments (Seghier, 2013). Combined studies of EEG/fMRI might be needed to understand the contribution of the area spanning posterior superior and middle temporal gyri and angular gyrus, to understand whether separate regions have a functional specificity with temporal relation in processing meaningful semantic information. It is clear however that the neural efficiency theory can not be used to explain our results, as we found increased rather than decreased neural activity in a area primary related to sentence processing. A possible explanation would be that the neural adaptability is not only selective to language processes, but also selective to which language processes, such as vocabulary (Prat and Just, 2011) but not high-level language abilities.

Previous studies by our group showed that activation in the right hemisphere (BA 47 and BA 22) correlated to language performance (Van Ettinger-Veenstra et al., 2010, 2012). In the experiments described here, we did not observe such right-hemispheric contribution of supporting language areas in direct relation to increased language ability performance. We observed an increased activation bilaterally in BA 45 that was congruency-related, but independent of language ability. Therefore we conclude that there seems not to be an interaction between semantic congruency related brain activation and high-level language performance, even though the semantic incongruent condition can be regarded as more demanding and evoking more neural activation. Further studies are needed to specify whether semantic incongruency evokes similar individual neural patterns in people with large vocabulary or high working memory as syntactic incongruency does, in terms of neural adaptability and recruitment of supporting language areas (Prat and Just, 2011).

Our investigation was limited in the sense that the paradigm was not optimized to enable application of the neural adaptability and neural efficiency theories, which may elucidate our findings and connect them to the above mentioned works on these theories. Also, our paradigm did not distinguish specific language processes that were tapped upon in our participants. We therefore believe that additional research is needed to differentiate the applicability of theories on brain adaptation and efficiency on different language processes. Moreover, we need to gain more understanding of how first language ability manifests itself dependent on congruency processing; which should include the exploration of the array of individual thresholds for neural adaptability in more detail.

## Conclusion

The activation increase of IFG bilaterally, emerging for incongruent compared to congruent sentence reading, suggests that the incongruent condition was more demanding than the congruent condition. However, we found no indications for a neural relation dependent on high-level language ability. Increased high-level language ability was characterized by increased left-hemispheric activation in the angular gyrus related to high language ability in general and emerged for both congruent and incongruent sentence reading. Despite the increased demands brought forth by the semantic incongruency condition, we can conclude that there seems not to be an interaction between semantic congruency related brain activation and high-level language performance.

## Author Contributions

HvE-V, AM, PL, TK and ME all contributed to designing the study. HvE-V and TK acquired the data, and HvE-V analyzed the data. HvE-V, AM, PL, TK and ME all interpreted the data. HvE-V and ME drafted the work, HvE-V wrote the manuscript, and HvE-V, AM, PL, TK and ME all contributed to revising the content, final approval of the manuscript and are accountable for the accuracy and integrity of the whole study.

## Conflict of Interest Statement

The authors declare that the research was conducted in the absence of any commercial or financial relationships that could be construed as a potential conflict of interest.

## Footnotes

^1^www.fil.ion.ucl.ac.uk/spm/software/spm8
